# Molecular Events for Promotion of Vancomycin Resistance in Vancomycin Intermediate *Staphylococcus aureus*

**DOI:** 10.3389/fmicb.2016.01601

**Published:** 2016-10-13

**Authors:** Qiwen Hu, Huagang Peng, Xiancai Rao

**Affiliations:** Department of Microbiology, College of Basic Medical Sciences, Third Military Medical UniversityChongqing, China

**Keywords:** vancomycin intermediate *Staphylococcus aureus*, vancomycin, genetic mechanisms, genotypes, molecular events

## Abstract

Vancomycin has been used as the last resort in the clinical treatment of serious *Staphylococcus aureus* infections. Vancomycin-intermediate *S. aureus* (VISA) was discovered almost two decades ago. Aside from the vancomycin-intermediate phenotype, VISA strains from the clinic or laboratory exhibited common characteristics, such as thickened cell walls, reduced autolysis, and attenuated virulence. However, the genetic mechanisms responsible for the reduced vancomycin susceptibility in VISA are varied. The comparative genomics of vancomycin-susceptible *S. aureus* (VSSA)/VISA pairs showed diverse genetic mutations in VISA; only a small number of these mutations have been experimentally verified. To connect the diversified genotypes and common phenotypes in VISA, we reviewed the genetic alterations in the relative determinants, including mutations in the *vraTSR, graSR, walKR, stk1/stp1, rpoB, clpP*, and *cmk* genes. Especially, we analyzed the mechanism through which diverse mutations mediate vancomycin resistance. We propose a unified model that integrates diverse gene functions and complex biochemical processes in VISA upon the action of vancomycin.

## Introduction

*Staphylococcus aureus* is a successful human pathogen because of its metabolic versatility and its ability to adapt to host defensive stress (Didelot et al., 2016). This pathogen can cause mild infections and life-threatening diseases, including skin and soft tissue infections, bacteremia, pneumonia, endocarditis, sepsis, and toxic shock syndrome (Dayan et al., 2016). Unfortunately, a licensed vaccine is unavailable for *S. aureus* infections. The optimal choice for treatment of *S. aureus* infections is the employment of antibiotics. However, antimicrobial resistance in *S. aureus* has become a major public health threat. The first antibiotic penicillin was discovered by Alexander Fleming in 1928 based on the susceptibility of *S. aureus*; subsequently, penicillin was clinically applied in a large scale during the early 1940s. After several years, the penicillin-resistant *S. aureus* (PRSA) was characterized in hospitals in the mid-1940s. PRSA strains usually carry a plasmid-encoded penicillinase, which can hydrolyze the β-lactam ring of penicillin to inactivate its antimicrobial activity. PRSA strains become pandemic by the early 1950s and were significantly controlled by the introduction of β-lactamase-resistant methicillin into clinic in 1959. However, the first methicillin-resistant *S. aureus* (MRSA) strain was quickly generated and isolated in 1961 then spreaded globally. MRSA strains are inherently resistant to virtually all β-lactam antibiotics, including penicillins, cephalosporins, and carbapenems. The emergence of MRSA resistance is the horizontal gene transfer of the *mecA* gene, which encodes an alternative penicillin binding protein 2a (PBP2a) with low-affinity to β-lactam antibiotics. With complicated evolution, MRSA has become a so-called “superbug” that has acquired resistance to multiple drugs, from penicillin/methicillin to quinolone and vancomycin (Nordmann et al., 2007).

**Figure 1:**
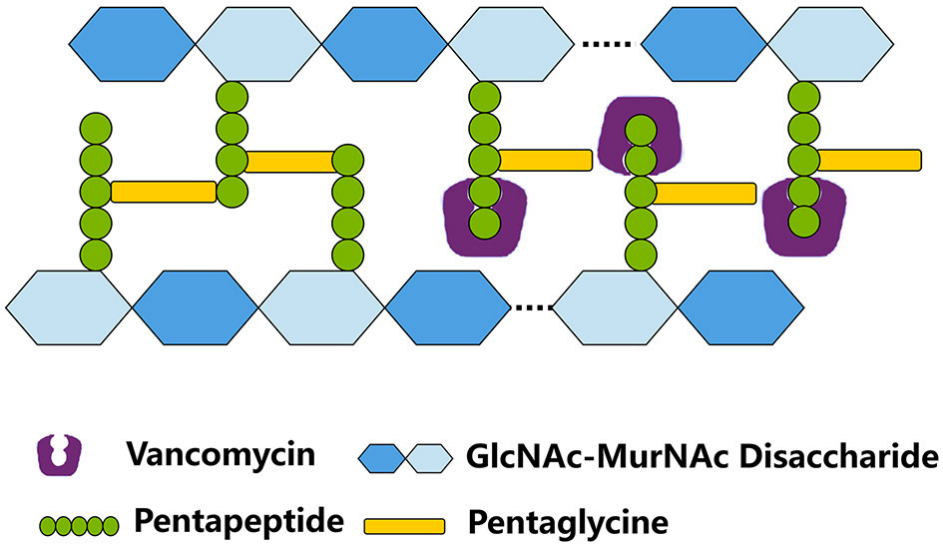
**The mode of action of vancomycin in *S. aureus***. By binding to the C-teminal D-Ala-D-Ala residues of the pentapeptide, vancomycin inhibits the cross bridge formation between pentapeptide and pentaglycine. GlcNAc, N-acetylglucosamine; MurNAc, N-Acetylmuramic acid.

**Figure 2:**
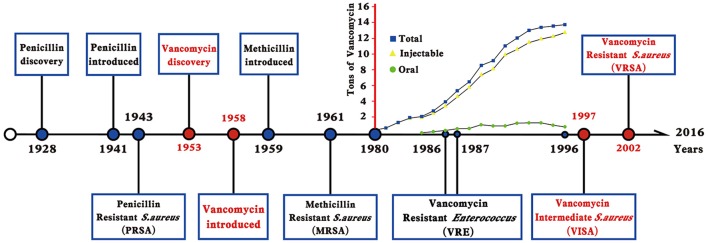
**Timeline indicates the year in which an event occurred or was reported**. The increased use of vancomycin in the USA, France, Italy, Germany, the United Kingdom, and the Netherlands was shown as tons of vancomycin in the y-axis, which was modified from reference Levine (2006).

Vancomycin is a cationic glycopeptide antibiotic derived from the organism *Amycolatopsis orientalis* (previously known as *Streptomyces orientalis* or *Nocardia orientalis*). Vancomycin was first discovered by Edmund Kornfield (working at Eli Lilly) in 1953 and approved by the Food and Drug Administration of the United States in 1958 because of the rapid development of penicillin resistance by staphylococci (Levine, 2006). However, due to the ototoxicity and nephrotoxicity caused by the presence of impurities and the development of other effective antibiotics, vancomycin was relegated to a second-line antibiotic in the 1960s and 1970s (Moellering, 2006). Vancomycin kills bacteria by binding to the C-teminal d-Ala–d-Ala residues of the peptidoglycan precursor lipid II in the cytoplasmic membrane to form a stable, non-covalent complex, which prevents the use of the precursor for cell wall synthesis (Figure 1). The steric hindrance imparted by vancomycin may further inhibit the glycosyltransferase and transpeptidase activities of penicillin-binding proteins (PBPs) (Kahne et al., 2005). Clinically, vancomycin is used for *S. aureus* infections and for infections caused by *Streptococcus pneumoniae, Clostridium difficile, Enterococcus* species, and so on. The increasing burden of MRSA and other Gram-positive bacterial infections in hospitals led to the increasing use of vancomycin worldwide since the 1980s (Levine, 2006). From 1980s to present, vancomycin is one of the last remaing antibiotics to which most of the MRSA and other multiple drug-resistant Gram-positive bacteria were still reliably susceptible. Moreover, vancomycin is used to treat osteomyelitis, bacteremia, and endocarditis empirically or when MRSA is deemed a possible cause (Rubinstein and Keynan, 2014). However, vancomycin-resistant *Enterococcus* (VRE) was first reported in 1986 in Europe then in the USA in 1987 (Murray, 2000) (Figure 2). *S. aureus* clinical isolates with reduced vancomycin susceptibility, such as the vancomycin-intermediate resistance *S. aureus* (VISA) strain Mu50 (MIC = 8 μg/mL) and the heterogeneous VISA (hVISA) strain Mu3 (MIC = 3 μg/mL), were first reported in Japan in 1997 (Hiramatsu et al., 1997a,b) then reported globally. VISA usually exhibits a low level of resistance, as defined by a vancomycin MIC from 4 to 8 μg/mL, although laboratory-derived VISA strains with vancomycin MICs of 32–100 μg/mL were achieved by *in vitro* mutagenesis (Berscheid et al., 2014; Ishii et al., 2015). Furthermore, the first vancomycin-resistant *S. aureus* (VRSA) isolate MI-1, with an MIC of 128 μg/mL, was recovered in 2002 from the foot wound of a diabetic patient who had received long-term vancomycin therapy and also had a VRE isolate.

VRSA is a rare, multidrug-resistant bacterial strain of public health concern. Since its emergence in the USA in 2002, 36 VRSA strains have been reported, 14 from the USA (Limbago et al., 2014; Walters et al., 2015), 16 from India, 3 from Iran, 1 from Pakistan (Moravvej et al., 2013), 1 form Portugal (Melo-Cristino et al., 2013), and 1 from Guatemala (Antony, 2014). VRSA arises when vancomycin resistance genes (e.g., the *vanA* operon, which codes for enzymes that result in modification or elimination of the vancomycin-binding site) from VRE are transferred to *S. aureus.* The *vanA* gene cluster is often located in the plasmid-borne transposon Tn1546, which can transfer from vancomycin-resistant *Enterococcus faecalis* to *S. aureus* and produce a VRSA isolate (Zhu et al., 2013). The mechanism for VRSA resistance is well-characterized. The coded product enables VRSA to replace the d-Ala–d-Ala terminal dipeptide with d-Ala–d-Lac dipeptide, thereby altering the binding target of vancomycin and often mediating high level resistance to vancomycin. Although VRSA may have been underestimated (Moravvej et al., 2013), the limited cases of VRSA suggest that *vanA*-mediated vancomycin resistance is significant but has not evolved or quickly spread; this trend is probably caused by the high fitness cost imparted by *vanA-*type transposon (Foucault et al., 2009). By contrast, VISA or hVISA isolates have been characterized from many countries around the world, including United States, France, Australia, Scotland, Brazil, Japan, South Korea, China, and other countries (Howden et al., 2010; Zhang et al., 2013). A retrospective study found that hVISA emerged before the introduction of vancomycin in Japan in 1990 (Yamakawa et al., 2012). Two other retrospective studies also revealed that the emergence of hVISA or VISA isolates in the USA or in Europe can be dated back to the mid or late 1980s (Rybak et al., 2005; Robert et al., 2006). Pulsed field gel electrophoresis and multilocus sequence typing found that VISA strains were not clonal. Most VISA were reported to evolve from hospital associated MRSA; mainly from clonal complex 5 or 8, in particular ST5 and ST239, however, the community associated MRSA clone USA300-derived and methicillin-susceptible *S. aureus* (MSSA)-derived VISA are also reported (Pillai et al., 2009; Gardete et al., 2012). In recent years, many studies also revealed that there is a vancomycin MIC creep in many countries but not consistently observed in different areas (Sader et al., 2009; Reynolds et al., 2012; Zhuo et al., 2013). The hVISA strains account for up to 26% of MRSA isolates in Japan (Schwaber et al., 2003), while in an Australian institute, the rate of hVISA is almost 50% (Horne et al., 2009). The high rate of hVISA paved the way for the evolution of VISA.

Aside from the vancomycin-intermediate phenotype, VISA strains exhibit common characteristics, such as thickened cell walls, reduced autolysis, and attenuated virulence (Howden et al., 2011). Understanding the molecular mechanisms for reduced vancomycin susceptibility in VISA has been rapidly moved forward by whole genome sequencing of vancomycin-susceptible *S. aureus* (VSSA)/VISA pairs or series (Howden et al., 2011). The mechanisms underlying VISA resistance may be complicated; diverse mutations occurred in VISA have been identified, but only a small number of mutations in several genes have been experimentally verified. In this review, we summarize the phenotypic features associated with VISA and thoroughly catalog the diverse mutations in VISA, especially those that have been experimentally characterized. Specifically, we reviewed and analyzed four categoris of genetic mutations occurred in VISA, including mutations in cell wall synthesis, hydrolysis, or remodeling genes, in metabolic genes, in transcription regulatory genes, and in post-translational modification genes. Based on these analyses, we connected the genetic mutations in different genes, biochemical functions of the cognate proteins, and key metabolic pathways that contribute to the promotion of vancomycin resistance in VISA.

## Common features of VISA

VISA exhibits several common phenotypic changes compared with VSSA; these features include increased cell wall thickness, reduced autolysis, decreased activity of the staphylococcal global regulator Agr, reduced lysostaphin susceptibility, and changes in cell wall teichoic acids (Nannini et al., 2010).

### Thickened cell walls with low cross-linking

Bacterial cell walls play important roles in the protection of viable cells, as well as the infectivity and pathogenicity of Gram-positive and Gram-negative bacteria. Staphylococcal cell walls are composed of murein, teichoic acids, and wall-associated surface proteins, with a thickness of ≈20 nm (Dmitriev et al., 2004). The stress-bearing murein of *S. aureus* consists of glycan strands, which are composed of *N*-acetylglucosamine (GlcNAc) and *N*-acetylmuramic acid (MurNAc) residues that form β-(1–4)-linked disaccharide repeating units. The carboxyl group of each MurNAc residue of the murein is amidated by the stem pentapeptide l-Ala–d-Gln–l-Lys–d-Ala–d-Ala; a pentaglycine bridge branches with the ε-amino group of the l-Lys residue of pentapeptide and the carboxyl group on the d-Ala terminus of the other pentapeptide to provide a high degree of murein cross-linking. The process of murein cross-linking is catalyzed by PBP2/PBP4 proteins.

Mu50 was the first VISA isolated from the purulent discharge at the sternal incision site of a 4 month-old male infant who underwent heart surgery for pulmonary atresia; its cell wall appeared to be twice as thick as that of control strains under an electron microscope (Hiramatsu et al., 1997b). The thickness of the cell wall was associated with the vancomycin resistance in VISA. Cui et al. (2003) demonstrated that cell wall thickening is a common feature of VISA isolates. Although the general principle of murein structural organization seems simple, the muropeptide composition of the staphylococcal cell wall appears very complex because the catalysis of murein cross-linking with PBPs is dose-dependent and often produces a distinctive degree of murein cross-linking. Hanaki et al. (1998) found that the incorporation of ^14^C-lablled GlcNAc into the cell wall and the intracellular murein monomer precursor was increased in Mu50; the activated cell wall synthesis and the resulting thickened cell walls might be a prerequisite for vancomycin resistance in VISA. Sieradzki and Tomasz (2003) revealed that VISA isolates in the JH serial strains produced thickened cell walls and upregulated the PBP2 protein level, similar to a Chinese VISA XN108 (Zhang et al., 2013). However, the protein level of PBP4 was drastically reduced, which decreased the cross-linking of VISA cell walls.

The thickened cell wall and decreased murein cross-linking were proposed to be significant for vancomycin resistance in VISA. The actual target of vancomycin is the free d-Ala–d-Ala terminal of the pentapeptide. The last d-Ala should be hydrolyzed by PBP4, and the pentaglycine of a murein monomer could be branched by PBP2 to finish the cross-linking. The cell wall of *S. aureus* usually has ≈20% of its peptidoglycan components that are not crosslinked, which produced an appropriate amount of free d-Ala–d-Ala residues in the cell wall. The thickened cell wall of a VISA usually has decreased cross-linking in its peptidoglycan, which will increase the amount of free d-Ala–d-Ala residues that allow the “capture” of more vancomycin molecules to protect VISA from the antibiotic (Sieradzki and Tomasz, 2003). Furthermore, the Gln-non-amidated murein monomer is increased in VISA, which is a poor substrate for PBPs but has a greater binding affinity for vancomycin than Gln-amidated muropeptide (Cui et al., 2000). Vancomycin has to pass through the peptidoglycan layers to reach the lipid II-bound muropeptide, which accumulates in the division septum. Thus, most vancomycin molecules might be trapped by the free d-Ala–d-Ala residues in the thickened cell walls of VISA. The cell wall thickness and vancomycin resistance are strongly correlated (Cui et al., 2000). Fluorescence microscopy shows that VISA strains can trap more vancomycin molecules than VSSA strains, thus less vancomycin molecules can arrive at the active cell wall synthesis site (Pereira et al., 2007). The thickened cell wall of Mu50 consumes more than 2.8 times of vancomycin than VSSA (Cui et al., 2000). After binding with vancomycin molecules, the structure of the outer layers of VISA thickened peptidoglycan is more compact to prevent further penetration of vancomycin molecules. This so-called “vancomycin clogging effect” might also contribute to vancomycin resistance in VISA (Cui et al., 2006).

### Decreased autolysis

Autolysis occurs in a wide variety of bacteria and is catalyzed by their own autolytic enzymes to cleave specific bonds in the bacterial cell wall peptidoglycan. In *S. aureus*, several muramidases and amidases, such as lysostaphin, AltA, and LytM, participate in autolysis. The autolytic activity could be determined by whole-cell assays in 0.05 M Tris–HCl (pH 7.2) containing 0.05% Triton X-100 or purified cell wall assays to retain the autolytic activities of the crude cell walls (Utaida et al., 2006). VISA strains often express reduced autolytic activity vs. that of VSSA (Howden et al., 2010). Mu50 was shown to be deficient in autolytic activity when whole-cell assays were conducted (Utaida et al., 2006). Correspondingly, the expression level of autolysins was down-regulated in VISA. The decreased autolysis in VISA may contribute to the cell wall thickening, thereby preventing vancomycin from reaching its action site.

### Decreased virulence

An *S. aureus* strain may suffer severe fitness cost upon developing resistance to certain antibiotics, such as resistance to ciprofloxacin or gentamicin; this fitness cost could be a disadvantage for its pathogenesis or clonal spreading in a given region (Shang et al., 2016). Several animal models were used to evaluate the virulence of VISA. In *Galleria mellonella*, a model insect, clinical VISA isolates have reduced virulence compared with their parental strains (Peleg et al., 2009; Howden et al., 2011). Furthermore, a VISA isolate showed reduced infectivity and rapid blood clearance compared with its parental strain in a rat endocarditis model (Majcherczyk et al., 2008). Another VISA isolate had attenuated virulence and lost the capacity to generate liver abscesses and tissue necrosis in a mouse sepsis model (Cameron et al., 2012). *S. aureus* produces tens of virulence factors for pathogenesis; VISA transcriptomic data were generally consistent with decreased virulence (Gardete et al., 2012). Hattangady et al. (2015) demonstrated that genes encoding secreted proteases and hemolysins were generally downregulated in two VISA strains vs. their progenitor VSSA strains. A VISA strain named SG-R, belonging to the MRSA clone USA300, showed massive downregulation in the transcription of virulence determinants, including seven of the major virulence genes of *S. aureus* (Gardete et al., 2012). Importantly, the transcription level of a virulence gene was restored when the vancomycin-resistant isolate SG-R was phenotypically “converted” to vancomycin susceptibility by the genetic complementation experiments (Gardete et al., 2012). These data indicate that the decreased virulence in VISA is associated with the same mutated gene responsible for the reduced vancomycin susceptibility. VISA strains with decreased or attenuated virulence fail to cause acute infections; however, decreased or attenuated virulence may represent a “stealth” strategy to evade host immune surveillance and promote clinical persistence (Gardete et al., 2012).

## Diverse mutations identified in VISA by whole genome sequencing

The molecular mechanisms for promotion vancomycin resistance in VISA are attractive because of the widespread incidence of VISA globally. The best way to identify the genetic mutations in VISA is the whole genome sequencing of carefully selected isogenic VSSA/VISA pairs or series from the clinic or derived from the laboratory. Table 1 shows the mutations identified by whole genome sequencing in hVISA/VISA strains that may be responsible for the reduced vancomycin susceptibility as compared with their progenitor VSSA. Between the VISA strain JH9 and the VSSA strain JH1, the JH9 strain had 35 point mutations in 31 genes (Mwangi et al., 2007). In the laboratory, a VISA could be obtained by step-wise selection with increasing concentrations of vancomycin in the culture media to exhibit a vancomycin MIC level of >32 μg/mL, such as VC40 [14], to the VRSA phenotype. Compared with its parent strain RN4220Δ*mutS*, VC40 had 79 mutations in 75 genes (Berscheid et al., 2014), thereby suggesting that the mutations in *S. aureus* genes may have cumulative effects that contribute to the VISA phenotype. Most studies usually reveal no more than 10 mutations in isogenic VSSA/VISA pairs (Table 1). However, one pair revealed that only one mutation (WalR-K208R) is enough to convert a VSSA to VISA (Howden et al., 2011). From the same parent VSSA strain JH1, two *in vitro* selected VISA isolates JH1R_1_ and JH1R_2_ have different mutations when compared with each other, which also differed from the *in vivo* VISA isolate JH2 (Vidaillac et al., 2013). These studies demonstrated that multiple pathways are involved in the generation of VISA from VSSA. However, the diverse genetic mutations that contribute to the altered cell wall structures and increased vancomycin resistance in VISA need to be experimentally investigated.

**Table 1:** **Mutations identified in hVISA/VISA strains comparied with the progenitor VSSA by whole genome sequencing**.

**VSSA/hVISA/VISA pairs or series**	**Vancomycin resistant level (μg/mL)**	**Number of mutated genes**	**Potential important mutations linked to VISA**	**Year**	**References**
JH1/JH2/JH5/JH6/JH9	1/4/6/8/8	7/8/16/31	VraT-H164R, RpoB-D471Y+A473S+A477S+E478D RpoC-E854K, PrsA-ΔC	2007	Mwangi et al., 2007
Mu3/Mu50	2/8	17	GraR-N197S, RpoB-H481Y, Fdh2-A297V, Sle1-Δ67aa	2008	Neoh et al., 2008
Mu50Ω/Mu50	0.5–1/6–7	10	GraR-N197S, VraS-234ΔC	2009	Cui et al., 2009
N315ΔIP/H14	1/2	1	VraS-S329L	2009	Katayama et al., 2009
ISP794/AR376	2/4	3	Stp1-Q12ΔC, VraS-G45R, YjbH-K23ΔC	2011	Renzoni et al., 2011
N315LR5P1/LR5P1-V3	1.5/4	2	WalK-ΔQ371, ClpP-ΔN	2011	Shoji et al., 2011
JKD6000/JKD6001	1/4	7	WalR-A96T	2011	Howden et al., 2011
JKD6004/JKD6005	2/4	1	WalR-K208R	2011	Howden et al., 2011
JKD6009/JKD6008	2/4	10	WalK-G223D, GraS-T136I	2011	Howden et al., 2011
JKD6021/JKD6023	1/4	8	WalK-V268F	2011	Howden et al., 2011
JKD6052/JKD6051	1/4	7	RpoB-H481Y, SarR-A68T	2011	Howden et al., 2011
SG-S/SG-R	1.5/3	5	VraT-Y220C, YycH-A165D, VraG-G551E	2012	Gardete et al., 2012
A5937/A5940	1.5/4	6	Stp1-113ΔC, H481Y	2012	Cameron et al., 2012
A6264/A6226	2/3	13	DltA-S38R, ArlS-38ΔC	2012	Cameron et al., 2012
A6300/A6298	2/4	8	Drp35-N83S	2012	Cameron et al., 2012
A9635/A9636/A9637/A9638/A9639	1/1/2/3/4	1/2/5/4	VraT-N74D, VraG-A580V	2012	Cameron et al., 2012
A8117/A8118/A8392	1/4/8	3/5	WalK-R263C-S273N, WalK-ΔQ371, TcaR-I69S-K95N, RpoB-S1052L	2012	Cameron et al., 2012
VSSA-A1/VISA-A2	1/8	6	Stp1-E18D19-duplication	2012	Passalacqua et al., 2012
JH1/JH1R_1_	1/3	4	WalK-G223D	2013	Vidaillac et al., 2013
JH1/JH1R_2_	1/3	1	RpoB-R484C+N641K	2013	Vidaillac et al., 2013
Mu3/Mu3 derived 45 VISA isolates	3/6–12	1–4 mutations affecting a total of 48 genes	BPB4-S140N, TarO-P94L, Cmk-A20G, etc.	2013	Matsuo et al., 2014
D32/D52/D56/D83/D90/D109	Not shown	1/1/4/6/4	WalK-Q369R, WalK-M220I, VraG-ΔL294N295	2014	Van Hal et al., 2014
109/2482	1.5/3	11	RpoB-A477V+S529L, AgrC-L193ΔC	2014	Chen et al., 2014
RN4220Δ*mutS*/VC40	1.5/64	75	VraS-L114S+D242G, WalK-I544M, MprF-H224Y, RpoD-D201N	2014	Berscheid et al., 2014
MW-2/SV-1	2/16 or 1/16	5	WalK-G223D, TarO-frameshift	2015	Hu et al., 2015
CH1/CH2/CH3/CH4/CH5/CH6/CH7	2/2/2/3/3/3/4	0/1/3/2/2/2	YycH-ΔC, MprF-S295L	2015	Chen et al., 2015
13136p−m+/13136p−m+V5/13136p−m+V20	1/8/16	8/9	Stp1-A143G, TarO-L670F	2015	Hattangady et al., 2015
8 MR/VR pairs	1–2/8–32	50–172 in different pairs	Diverse mutations in GraS, RpoB, RpoC, WalK and etc.	2015	Ishii et al., 2015

ΔN, N-terminal deletion; ΔC, C-terminal deletion.

## Experimentally verified genetic mutations in certain genes responsible for VISA evolution

Although hundreds of single nucleotide polymorphisms (SNPs) were discovered in VISA as compared with VSSA after the whole genome sequencing of VSSA/VISA pairs or series, the experimentally verified genetic mutations in certain genes responsible for VISA evolution are still limited. Two methods are usually used to functionally verify the role of a certain mutation for the promotion of vancomycin resistance in VISA. First, the mono- or bi-directional allelic exchange experiment replaces the normal allele in VSSA with the mutated allele from VISA or replaces the mutated sites in VISA with the normal allele from VSSA; subsequently, the results are used to evaluate whether the allele swapping is responsible for vancomycin resistance. Some studies also conduct whole genome sequencing of the allele-swapping strains to exclude other mutations during experiments (Howden et al., 2011). The pKOR1 plasmid is usually used in these allele-swapping experiments because it has a counter-selection marker and can be used to generate multiple allele swapping events (Bae and Schneewind, 2006). For VISA clinical isolates that cannot be transformed, the mutations are reconstituted in well-defined laboratory strains, such as N315ΔIP (Katayama et al., 2016). The constructed strains are subject to vancomycin MIC determination, cell wall thickness measurement with electron microscopy, and other tests to verify and explore the mechanisms of gene mutation in vancomycin resistance (Cameron et al., 2012). Another genetic method is plasmid complementation with wild type or mutated genes in VSSA or VISA isolates; however, the copy number of plasmids cannot be precisely controlled, and the results of complementation are usually not consistent with the allele swapping results (Neoh et al., 2008; Matsuo et al., 2011). Regardless, plasmid complementation is easy and labor-saving; thus, this approach is still widely used for the verification of loss-of-function mutations (Gardete et al., 2012; Matsuo et al., 2014).

Allelic exchange techniques or complementation methods have experimentally verified the four categories of genetic mutations in VISA as summarized in Table 2. The first category includes the genetic mutations in cell wall synthesis, hydrolysis, or remodeling genes, including *sle1* and *msrR* (Katayama et al., 2016). The second group includes the genetic mutations in metabolic genes, including *cmk* and *fdh2* (Matsuo et al., 2014; Katayama et al., 2016). The third category contains the genetic mutations in transcription regulatory genes, including *yvqF*/*vraT*-*vraSR, graSR, walKR*, and *rpoB* (Howden et al., 2014). The fourth group has the genetic mutations in post-translational modification genes, including *stp1* and *clpP* (Shoji et al., 2011; Cameron et al., 2012).

**Table 2:** **Experimental verified mutations in VISA**.

**Target**	**Year**	**Mutation Sites**	**Parental strain**	**Vancomycin MIC (μg/mL) changes**	**Methods**	**References**
VraTSR	2009	VraS-S329L	N315ΔIP	1 → 2	pKOR1 mediated allele swapping	Katayama et al., 2009
	2012	VraT-Y220C	SG-R	3 → 1.5	pGC2 mediated complementation with wild type VraT	Gardete et al., 2012
	2012	VraS-234Δ	SG-rev	1 → 3	pGC2 mediated complementation with wild type VraS	Gardete et al., 2012
	2014	VraS-L114S+D242G	NCTC8325	1.5 → 4	Temperature-sensitive shuttle vector pMAD mediated allele swapping	Berscheid et al., 2014
GraSR	2008	GraS-T136I	JKD6009	2 → 6	pKOR1 mediated allele swapping	Howden et al., 2008
	2008	GraR-N197S	Mu3	2 → 4	pYT3 mediated overexpression	Neoh et al., 2008
	2011	GraS-T136I	JKD6009	1.5 → 2	pKOR1 mediated allele swapping	Howden et al., 2011
WalKR	2011	WalK-G223D	JKD6009	1.5 → 3	pKOR1 mediated allele swapping	Howden et al., 2011
	2011	WalR-K208R	JKD6004 or JKD6005	1.5 → 4 or 4 → 1.5	pKOR1 mediated allele swapping	Howden et al., 2011
	2011	WalK-ΔQ371	LR5P1	1.5 → 3	pKOR1 mediated allele swapping	Shoji et al., 2011
	2015	WalK-G223D	MW2	2 → 4	pBTs mediated allelic replacement, pBTs is derived from pBT2 and pKOR1	Hu et al., 2015
ClpP	2011	ClpP-ΔN	LR5P1	1.5 → 2	pKOR1 mediated allele swapping	Shoji et al., 2011
Stp1	2012	Stp1 deletion	A5937	1.5 → 3	pKOR1 mediated gene deletion	Cameron et al., 2012
	2012	Stp1-E18D19 duplication	Strain A2	6–8 → 3	pOS1-P*lgt* mediated wild type Stp1 complementation	Passalacqua et al., 2012
Cmk	2014	Cmk-A20G, CmK-T(-13)A	Mu3	2 → 8, 3 → 8	pKOR1 mediated allele swapping	Matsuo et al., 2014
	2014	Cmk-A20G, CmK-T(-13)A	Mu3p27V6–10	8 → 2, 8 → 3	Introduce the pND50-*cmk* plasmid into the VISA isolates	Matsuo et al., 2014
VraS+GraR	2009	VraS-I5N+GraR-N197S	Mu50Ω	4 → 6	pKOR1-mediated allele swapping	Cui et al., 2009
GraS+WalK	2011	GraS-T136I+ WalK-G223D	JKD6009	1.5 → 4	pKOR1-mediated allele swapping	Howden et al., 2011
GraR+RpoB	2011	GraR-N197S+RpoB-H481Y	Mu3	2 → 6	pKOR1-mediated allele swapping	Matsuo et al., 2011
WalK+ClpP	2011	WalK-ΔQ371+ ClpP-ΔN	LR5P1	1.5 → 4	pKOR1 mediated allele swapping	Shoji et al., 2011
VraS+Stp1+YjbH	2011	VraS-G45R+Stp1-Q12ΔC+YjbH-K23ΔC	ISP794	2 → 4	Plasmid mediated gene replacement and bacteriophage transduction mediated triple mutant construction	Renzoni et al., 2011
VraS+GraR+RpoB+Fdh2+Sle1+MsrR	2016	VraS-S329L+GraR-N197S+RpoB-H481Y+Fdh2-A297V+Sle1-Δ67aa+MsrR-E164K	N315ΔIP	1 → 12	pKOR1 mediated allele swapping	Katayama et al., 2016

The vancomycin MIC levels determination methods are not consistent with each other in different studies.

### Mutated genes for cell wall synthesis, hydrolysis, and teichoic acid synthesis

Accelerated cell wall synthesis and decreased autolysis are alternative pathways for a thickened cell wall. Sle1 is the hydrolase of *N*-acetylmuramyl-l-alanine amidase for peptidoglycan biosynthesis and is involved in the cell wall separation of *S. aureus* (Kajimura et al., 2005). Six mutations were found in the VISA strain Mu50, but not in the VSSA strain N315ΔIP; one of these mutations is *sle1* (Δ67aa). The deleted 67-aa polypeptide was localized to the LysM domain; thus, *sle1* (Δ67aa) is a loss-of-function mutation. The introduction of *sle1*(Δ67aa) into N315ΔIP drastically decreased the autolytic activity and converted the induced cell wall thickening to constitutive cell wall thickening (Katayama et al., 2016). Loss-of-function mutations in cell wall hydrolysis genes, such as *sle1*, can directly contribute to vancomycin resistance in VISA.

MsrR is a member of the LytR–CpsA–Psr (LCP) family, which attaches wall teichoic acid (WTA) to the peptidoglycan layer (Chan et al., 2013). The deletion of *msrR* reduced WTA attachment and increased the cell size and aggregation (Hübscher et al., 2009). WTA controls staphylococcal autolysis by preventing the binding of autolysin to the assembled cell wall, but not to the septum (Schlag et al., 2010). Expression of *msrR* is increased by ≈four-fold upon vancomycin treatment (Gardete et al., 2006). Overexpression of *msrR* in the VSSA strain N315 reduced vancomycin and teicoplanin susceptibility (Cui et al., 2005). The *msrR* (E164K) mutation is one of six mutations identified in Mu50. The introduction of this mutation into N315ΔIP increased the vancomycin resistance (Katayama et al., 2016). The MsrR (E164K) mutation may promote the attachment of WTA to the peptidoglycan layer and further prevent the binding of autolysin to the assembled cell wall, thereby decreasing cell wall autolysis and resistance to vancomycin. However, whether the MsrR (E164K) mutation is a gain-of-function mutation or a loss-of-function mutation is still not resolved.

WTA is a highly negative charged polymer; the downregulated production of WTA may decrease the negative charge of the cell surface, thereby also contributing to vancomycin resistance via electrical repulsion. The PBP4 is required for the highly cross-linked peptidoglycan synthesis. PBP4 is localized at the division septum; however, in the WTA synthesis gene *tagO* deletion mutant, PBP4 was dispersed on the entire cell membrane and could not function properly; thus, the degree of cross-linking is decreased (Atilano et al., 2010). Furthermore, WTA also controlled autolysin activity by influencing its localization (Schlag et al., 2010). The loss of all the WTA caused by *tagO* gene deletion enhanced autolysis (Schlag et al., 2010); however, the inhibition of the late WTA synthesis by Targocil decreased autolysis and strongly induced cell wall stress stimulon genes, including *pbpB* and *fmtA* (Campbell et al., 2012). To obtain a more comprehensive view of hVISA-to-VISA conversion, 45 high-level VISA isolates were selected by exposing Mu3 and its related strains to 6 μg/mL vancomycin. Among the 45 VISA isolates, 32 have a single mutation in 20 genes, thereby indicating that these genes are directly involved in vancomycin resistance. Among the 32 single mutations, six mutations were identified in the *tarO, tarA*, and *tarL* genes (Matsuo et al., 2014). A frame shift insertion mutation in *tarO* (*llM*) was also discovered in the laboratory MW-2-derived VISA isolate SV-1 (Hu et al., 2015). These studies collectively indicate that mutations or the regulation of the WTA can contribute to vancomycin resistance in VISA.

The lipoteichoic acid (LTA) is another negatively charged polymer, which is linked to the cell membrane via a diglucosyl–diacylglycerol (Glc2-DAG) linkage. In the LTA synthesis pathway, glucose 6-phosphate (G6P) is converted to α-G1P by PgcA, then α-G1P is activated by GtaB to generate UDP-glucose; YpfP catalyzes the progressive addition of glucose to diacylglycerol (DAG) from UDP-glucose to yield the LTA anchor Glc2-DAG, which is flipped out by the LtaA protein. The loss of function of PgcA, GtaB, and YpfP did not inhibit LTA formation but altered the anchoring site and chain length of LTA (Gründling and Schneewind, 2007), thereby influencing the cell wall characteristics. In Δ*ypfP*, the autolysis activity is decreased compared with the wild type (Fedtke et al., 2007). Interestingly, one Mu3-derived VISA isolate has a C-terminal deletion of GtaB, which lacks the catalytic core of GtaB. This loss-of-function mutation in GtaB blocks the G6P to α-G1P conversion; subsequently, G6P might be redistributed to the peptidoglycan precursor synthesis. The bacterial two-hybrid approach revealed that YpfP and LtaA can interact with numerous proteins involved in cell division, peptidoglycan synthesis, or cell wall modification, including FtsA, FtsW, PBP2, PBP4, and DltD (Reichmann et al., 2014). Overall, mutations in the synthesis of peptidoglycan, WTA, and LTA or the remodeling processes can directly or indirectly contribute to vancomycin resistance in VISA.

### Mutated genes involved in staphylococcal metabolism

Cell wall synthesis consumes high amounts of substrate and energy. To build a thickened cell wall, the demand for cell wall biosynthetic precursors is increased in VISA, which requires the adjustment of cellular metabolism. VISA strains have impaired acetate catabolism (Nelson et al., 2007). Genetically distinct VISA strains are also associated with specific and reversible metabolic alterations (Alexander et al., 2014). The abundance of six metabolites from the urea cycle, the pentose phosphate pathway, and the TCA cycle was altered in the JH series and the SG series of VISA isolates (Alexander et al., 2014). These altered metabolites are directly or indirectly linked to the biosynthesis of cell wall precursors. Mutations in metabolic genes can directly adjust cellular metabolism to support the cell wall synthesis.

The *cmk* gene product functions in the pyrimidine synthesis pathway. A decrease in Cmk activity is expected to increase UTP, which is required for the synthesis of a key peptidoglycan intermediate UDP-GlcNAc. Two mutated forms of the *cmk* gene have been introduced to Mu3, thereby converting the hVISA isolate Mu3 to VISA isolates. One mutant is the Cmk-A20G mutation; the other is a T-to-A mutation in the Shine-Dalgarno (SD) sequence of Cmk (Matsuo et al., 2014). The Cmk-A20G mutation decreased the Cmk activity, whereas a mutation in the SD sequence decreased the translation of Cmk. A decrease in the Cmk activity or Cmk protein levels probably caused increased vancomycin resistance by increasing the supply of cell wall biosynthesis intermediate UDP-GlcNAc (Matsuo et al., 2014). Cmk catalyzed the formation of cytidine diphosphate (CDP). CDP is required for DNA/RNA synthesis and the formation of the WTA synthesis precursor CDP-glycerol. Peptidoglycan and WTA production depend on the lipid carrier undecaprenyl phosphate; the Cmk mutation might suppress the WTA synthesis pathway and enhance peptidoglycan synthesis.

Fdh2 is a putative formate dehydrogenase. The introduction of an Fdh2-A297V mutation into N315ΔIP with the aforementioned Sle1(Δ67aa) converted the inducible cell wall thickening to constitutive cell wall thickening (Katayama et al., 2016). Therefore, mutations in metabolic genes can directly influence cell wall synthesis and contribute to vancomycin resistance in VISA. Several other mutated metabolic proteins have been identified in VISA isolates, including proteins involved in translation, nucleotide metabolism, amino acid biosynthesis, and so on. These proteins may also directly adjust the cell metabolism to support cell wall synthesis in VISA.

### Mutated genes for transcriptional regulation

Among the cell wall synthesis or hydrolysis genes, the metabolic genes are dynamically regulated by transcriptional factors and other regulators. Hundreds of genes (≈10% of the whole *S. aureus* genome) from several functional categories are differentially expressed in VISA and VSSA. Previous studies have directly identified mutations in transcriptional regulators that may be the key players of vancomycin resistance in VISA isolates. Most mutations in *vraTSR, graSR, walKR*, and *rpoB* have been experimentally verified to be responsible for promoting vancomycin resistance in VISA.

#### Mutation activated VraTSR system

VraSR is characterized as vancomycin resistance-associated because the VraSR operon is upregulated in the first reported VISA isolate Mu50 (Kuroda et al., 2000). The operon encompassing VraSR consists of four genes. The *orf1* gene is not required for cell wall stress stimulon, whereas VraT/YvqF–VraS–VraR is a three-component system that regulates cell wall stress stimulon (McCallum et al., 2011; Boyle-Vavra et al., 2013). VraTSR positively modulates the cell wall biosynthesis pathway in *S. aureus* (Kuroda et al., 2003). After induction by a cell wall synthesis inhibitor, VraR activates the *vra* operon and 46 other genes for the cell wall stress regulon; some of these genes, such as *pbpB* and *fmtA*, encode known or putative cell wall synthesis proteins (Gardete et al., 2006). The use of an IPTG-inducible promoter controlled *pbpB* revealed that the VraTSR system might directly sense a cell wall synthesis step catalyzed by PBP2 (Gardete et al., 2006). The consensus binding site of VraR has been deduced (5′-ACT-N3-AGT-3′ or 5′-TGA-N3-TCA-3′) (Belcheva et al., 2009). Chromatin immunoprecipitation showed that the promoters of *pbpB, murZ*, and *sgtB* could directly be binded by the VraR proteins, whereas direct binding was not possible with *fmtA* (Sengupta et al., 2012). Upregulation of VraSR and the VraSR-dependent cell wall stimulon is linked to the increased vancomycin resistance phenotypes of several clinical VISA isolates (Kuroda et al., 2000; McAleese et al., 2006; Kato et al., 2008, 2010). By contrast, deletion of the *vraSR* operon enhanced the sensitivity to vancomycin and other cell wall targeting antibiotics (McCallum et al., 2011; Boyle-Vavra et al., 2013).

The VraT-Y220C, VraS-I5N, VraS-S329L, and VraS-L114S-D242G mutations have been experimentally associated with the VISA phenotype. Whole genome sequencing of the susceptible SG-S, the resistant strain SG-R, and the spontaneous revertant strain SG-rev in an isogenic VISA-type series of ST8-USA300 isolates revealed that the VraT-Y220C mutation mediates the vancomycin-resistant phenotype, whereas a premature termination of the VraS at amino acid 234 reverts the vancomycin-resistant phenotype (Gardete et al., 2012). VraS has 347 amino acids but the histidine kinase domain is lost when shortened to 234 amino acids. These data suggest that VraT is a negative regulator of VraSR, and VraT-Y220C is a loss-of-function mutation. Complementation of the VISA isolate with wild-type *vraT* can revert the vancomycin-resistant phenotype, whereas complementation of the reverted VSSA isolate with wild-type VraS can promote the vancomycin-resistant phenotype (Gardete et al., 2012).

The introduction of the VraS-I5N or VraS-S329L mutation into a VSSA isolate increased vancomycin resistance (Cui et al., 2009; Katayama et al., 2016). The reconstitution of the VraS-L114S-D242G allele into the VSSA strain NCTC8325 increased resistance to vancomycin (Berscheid et al., 2014). Furthermore, the cell wall stress regulon controlled by VraSR is upregulated in these VISA isolates. All these studies indicate that mutations in the VraTSR system cause its activation and the upregulation of the cell wall stress regulon, thereby increasing cell wall synthesis and vancomycin resistance in VISA.

#### Mutation activated GraSR system

The GraSR system is a two component system (TCS) associated with glycopeptide resistance (Cui et al., 2005). The GraSR proteins interact with GraX and the VraFG ABC transporter to form a five-component system for cationic antimicrobial peptide (CAMP) resistance (Falord et al., 2012a). The GraSR system is required for CAMP resistance, such as resistance to human defensins. Resistance to CAMPs is mediated by the d-alanylation of cell wall teichoic acids by the DltABCD enzymes and the MprF-dependent lysylination of phoshpatidylglycerol (Ernst and Peschel, 2011). GraR can self-dimerize; upon phosphorylation by GraS on the D51 residue, GraR binds to the promoters of *vraFG, dltABCD*, and *mprF* to initiate their transcription. A highly-conserved 10 base pair palindromic sequence (5′-ACAAATTTGT-3′) is the putative GraR-binding site, which is located in the upstream sequences of *mprF, dltABCD*, and *vraFG* genes.

The GraSR system is upregulated in VISA isolates (Cui et al., 2005). The deletion of GraSR or VraFG caused hypersensitivity to vancomycin, increased autolysis, and produced a more negative net surface (Meehl et al., 2007). The Mu50 strain has a GraR-N197S substitution; the introduction of this substitution on the plasmid pYT3 vector can convert the hVISA strain Mu3 to achieve high vancomycin resistance similar to that of the VISA strain Mu50 (Neoh et al., 2008). However, the GraR-N197S substitution on the pYT3 vector is a high-copy version; the introduction of a single copy of GraR-N197S into Mu3 did not increase vancomycin resistance to the level of VISA (Matsuo et al., 2011). Thus, the GraR N197S only has a marginal effect on vancomycin resistance. The GraR-N197S substitution and the RpoB-H481Y mutation can convert Mu3 to the full vancomycin resistance level of Mu50 (Matsuo et al., 2011). The GraS-T136I mutation can also increase the vancomycin resistance levels (Howden et al., 2008). However, GraS-T136I alone is not sufficient to convert VSSA to the full vancomycin resistance of VISA. The combination of GraS-T136I and WalK-G223D is required to achieve the full vancomycin resistance level of VISA (Howden et al., 2011). All these studies indicate that the activated GraSR system can increase vancomycin resistance by upregulating the *dltABCD* and *mprF* genes.

#### Mutation downregulating the WalKR system expression or activity contributed to vancomycin resistance in VISA

As the only essential TCS for the viability of *S. aureus*, the WalKR system connects cell wall biosynthesis with cell division. WalK has two transmembrane helices and a C-terminal kinase domain. The N-terminal Per–Arnt–Sim (PAS) domain may be involved in signal sensing. WalR controls the expression of its regulon by forming a head-to-head dimer of the receive domains, which is paired with a head-to-tail dimer of the winged helix-turn-helix motifs that bind to the tandem DNA repeats of the binding site (Dubrac et al., 2008). The potential consensus DNA recognition sequences of WalR consist of two hexanucleotide direct repeats (5′-TGT(A/T)A(A/T/C)-N5-TGT(A/T)A (A/T/C)-3′) (Dubrac et al., 2008). In VISA isolates, the expression levels of *altA, sle1, lytM*, and several genes encoding CHAP domain-containing proteins were down-regulated. The genes for AtlA, Sle1, LytM, and the CHAP domain-containing proteins are repressed by TCSs, such as LytSR and ArlSR, and positively regulated by the WalKR. Promoter analysis demonstrated that *atlA, sle1*, and *lytM* have the consensus sequence for WalR binding. Expression analysis by WalKR starvation or the constitutive form of WalR supported that *atlA, sle1*, and *lytM* are directly transcribed by the WalKR system in *S. aureus* (Dubrac et al., 2007; Delauné et al., 2012).

Two studies examined an IS256 insertion into the *walKR* promoter and revealed that the upregulation of *walKR* expression will decrease resistance levels to vancomycin, whereas the downregulation of *walKR* expression will increase the resistance levels of vancomycin (Jansen et al., 2007; McEvoy et al., 2013). Jansen et al. (2007) compared two VISA isolates SA137/93A (MIC = 8 μg/mL) and SA137/93G (MIC = 12 μg/mL) and found that SA137/93A has an IS256 insertion in the predicted promoter region. However, this IS256 insertion generates a potentially stronger promoter, which upregulated the *walKR* expression of SA137/93A; thus, the upregulation of WalKR produces low levels of vancomycin resistance (8 vs. 12 μg/mL), thereby indicating that *walKR* is a negative regulator of vancomycin resistance (Jansen et al., 2007). An IS256 insertion to the 5′-untranslated region (5′-UTR) of *walKR* reduced the expression of *walKR* by ≈50%; the downregulation of *walKR* will increase the vancomycin resistance level of VSSA isolates (McEvoy et al., 2013). The removal of IS256 could revert the VISA phenotype to VSSA, thereby confirming that the reduced expression of WalKR induced the VISA phenotype. These studies collectively suggest that the downregulation of *walKR* expression contributes to the development of vancomycin resistance in VISA.

Several studies examined the mutated forms of WalKR in vancomycin resistance of VISA. In a laboratory-derived VISA strain, Shoji et al. (2011) found that the *walK*ΔQ371 allele influences vancomycin resistance, but this allele is not sufficient for full vancomycin resistance. However, the *walKR* mutation was the most frequent in VISA strains. Genome sequencing of five VSSA/VISA pairs revealed that the VISA in four of these pairs carried mutations in the WalKR system (Howden et al., 2011). Subsequently, allelic replacement demonstrated that WalK-G223D with GraS-T136I could convert VSSA to full intermediate vancomycin resistance similar to VISA. The allelic replacement of WalR-K208R can also convert VSSA to full intermediate resistance to vancomycin (Howden et al., 2011). Howden et al. (2011) proposed that mutations in WalKR may downregulate its activity. SV-1 is a laboratory-derived VISA strain from CA-MRSA MW2 with the same WalK-G223D mutation as the clinically-isolated strain JKD6008 (Hu et al., 2015). The WalK-G223D mutation had decreased autophosphorylaton of WalK, thereby reducing the phosphorylation of the WalR response regulator and decreasing the binding activity of WalR to the *altA* promoter based on the electrophoretic mobility shift assay (Hu et al., 2015). Therefore, the downregulated expression or impaired activity of the WalKR system can mediate vancomycin resistance in VISA by downregulating the expression of autolysin genes.

#### Mutation altered the activity of RpoB

The *rpoB* gene encodes the β subunit of RNA polymerase, which is active in catalysis. Mutations in RpoB might influence the transcriptional activity of the whole RNA polymerase. The most extensively studied mutation of RpoB in VISA is the H481Y substitution. The introduction of a single copy of the GraR N197S into the Mu3 is not enough to achieve the vancomycin resistance level of the VISA isolate Mu50 (Matsuo et al., 2011). Targeted sequencing of the rifampicin resistance-determining region (RRDR) showed that Mu50 has an H481Y mutation at RpoB. Genetic swapping studies confirmed that the RpoB-H481Y mutation is responsible for the emergence of the VISA phenotype (Matsuo et al., 2011). The RpoB-H481Y mutation causes global transcriptional changes, which promote antimicrobial peptide resistance, with attenuated virulence in a murine bacterimia model (Gao et al., 2013). Mutations in other subunits of RNA polymerase such as RpoC or RpoD, have also been found in VISA isolates (Matsuo et al., 2014). The ST239-MRSA-III-t030 clone is prevalent in China; most ST239-MRSA-III-t030 strains harbor double substitutions in RpoB (H481N+L466S) that confer resistance to rifampicin, but not to vancomycin (Zhou et al., 2012; Shang et al., 2016). Therefore, the RpoB-H481N mutation may not confer vancomycin resistance similar to that of the RpoB-H481Y mutation; otherwise, a second mutation (L466S) could compensate for the effect of H481N on vancomycin resistance. This hypothesis is worthy of further investigation.

#### SarA/MgrA family proteins in VISA

The staphylococcal accessory protein A (SarA) family of global regulatory proteins control virulence, antibiotic resistance, cell wall synthesis, and other defensive pathways. SarA and MgrA are negative regulators of murein hydrolases (Ingavale et al., 2003). SarV is repressed by SarA and MgrA and is involved in autolysis (Manna et al., 2004). The *sarA* deletion mutant displayed enhanced resistance to vancomycin (Sun et al., 2012). Moreover, *mgrA* overexpression increased vancomycin resistance in *S. aureus* (Cui et al., 2005). An A68T mutation in SarR was identified in a VISA clinical isolate; however, this mutation has not been experimentally verified (Howden et al., 2011).

In the constitutive WalR mutant, the expression of *sarS, sarT*, and *icaR* was repressed by the WalKR system (Delauné et al., 2012). The *sarS* and *sarR* expression is also positively controlled by GraSR (Falord et al., 2012b). The *msaABCR* operon can positively regulate the expression of *sarA* to promote biofilm formation and enhance virulence while negatively regulating autolysis in *S. aureus* (Sambanthamoorthy et al., 2006). The inactivation of the *msaABCR* operon in three different VISA isolates increased the susceptibility to vancomycin and reduced the vancomycin-binding capacity (Samanta and Elasri, 2014). These studies collectively indicate that the SarA family proteins are crucial regulators in VISA.

#### CcpA and CcpE, potential regulators in VISA

The catabolite control protein A (CcpA) is the major regulator of carbon catabolite repression (CCR). In the presence of glucose or other preferred carbon sources, CcpA forms a complex with S46-phosphorylated Hpr protein. The CcpA–Hpr-S46(P) complex binds to the catabolite responsive element (cre) sequences of diverse genes to activate or repress their expression. The expression of *ccpA* is negatively self-regulated (Leiba et al., 2012). The expression of several genes is affected by *ccpA* inactivation in the absence of glucose, thereby indicating that the function of CcpA is not restricted to CCR and includes virulence and antibiotic resistance regulation (Seidl et al., 2009). CcpA can also regulate numerous virulence factors (Seidl et al., 2006). The expression of the urease operon is downregulated in Δ*ccpA* compared with the wild type, which is consistent with results of the urease activity assay (Seidl et al., 2009).

Transcription analysis revealed that the phosphorylation transfer system (PTS) of different carbon sources is usually influenced by antibiotic treatment or the *vraSR, graSR*, and *walKR* systems. The expression of PTS genes is directly controlled by the CcpA protein. CcpA represses the tricarboxylic acid (TCA) cycle, which is required for the full oxidation of glucose; CcpA also regulates the genes involved in the glycolytic pathway and metabolism (Seidl et al., 2009). Although the CcpA mutation was not observed in VISA isolates, the deletion of *ccpA* reduced teicoplanin resistance levels in a glycopeptide-intermediate *S. aureus* strain (Seidl et al., 2006). The altered central metabolism in VISA can also regulate CcpA activity, which can be fine-tuned by small molecule effectors, such as G6P and fructose 1,6-bisphosphate (FBP), which both enhance the DNA-binding activity of the CcpA–Hpr-S46(P) complex (Schumacher et al., 2007). However, the G6P and FBP levels significantly decreased in the Δ*stp1* strain, which has increased vancomycin resistance (Liebeke et al., 2010). Mutations in metabolic genes or the altered central metabolism in VISA isolates might control gene expression via the CcpA protein.

Catabolite control protein E (CcpE) can also affects the central metabolism as well as virulence. CcpE is the first positive regulator of TCA cycle reported in *S. aureus* by activating the expression of acotinase gene (*citB*) (Hartmann et al., 2013). In addition, CcpE positively regulate the expression of acetyl-CoA carboxylase (AAC) gene, while AAC catalyzes the committed step in fatty acid biosynthesis (Ding et al., 2014). On the other hand, CcpE negatively control the expression of many virulence genes (Ding et al., 2014). Intriguingly, CcpE is allosterically activated by the TCA cycle intermediate citrate (Ding et al., 2014). The altered central metabolism observed in VISA might control gene expression via the CcpE protein and vice versa, though no genetic mutations occured in CcpE have been observed in VISA isolates.

### Mutated genes for protein phosphorylation or degradation mediate vancomycin resistance in VISA

Aside from transcriptional control, the protein phosphorylation and degradation processes are widely used as regulatory mechanisms. The reversible phosphorylation and dephosphorylation of proteins by Stk1/Stp1 have important roles in diverse cellular processes of *S. aureus* (Ohlsen and Donat, 2010). Moreover, the ATP-dependent ClpP protease involved in intracellular proteolysis plays important roles in stress response, metabolism and cell wall synthesis (Frees et al., 2014). Mutations in *stp1* or *clpP* have been associated with vancomycin resistance in VISA.

#### Mutations in stk1/stp1

The Stk1 (PknB)/Stp1 (PP2C) pair is a eukaryotic-like Ser/Thr kinase/phosphatase system in bacteria. Stk1 and Stp1 are usually found to function together to regulate the reversible phosphorylation of substrates. Stk1 is also a substrate of Stp1 (Débarbouillé et al., 2009). In *S. aureus*, Stk1 is a membrane-bound protein, whereas Stp1 is a cytosolic protein. The N-terminal domain of Stk1 inside the cytoplasm is associated with kinase activity, whereas the C-terminal domain of Stk1 has three penicillin-binding protein and serine/threonine kinase-associated (PASTA) domains, which are hypothesized to respond to cell wall signals. Biophysical and structural studies revealed the direct interactions of the PASTA domain of Stk1 with muropeptides (Ruggiero et al., 2012). The deletion of *stk1, stp1*, or both can influence cell wall structure and antibiotic susceptibility. The *stp1* deletion mutant displayed a thickened cell wall and increased resistance to lysostaphin (Beltramini et al., 2009). Furthermore, the s*tk1* deletion mutant was more virulent than the parental strain in a murine cutaneous infection model (Tamber et al., 2010). By contrast, the *stp1* deletion mutant was avirulent in a mouse sepsis model (Cameron et al., 2012).

Three studies examined the role of protein phosphatase Stp1 in VISA isolates or in isolates with decreased susceptibility to teicoplanin (Renzoni et al., 2011; Cameron et al., 2012; Passalacqua et al., 2012). In the three studies, the loss of *stp1* function via the deletion of *stp1* or a premature stop codon increased the vancomycin and teicoplanin resistance levels. A two amino-acid insertion mutation at the highly conserved metal-binding domain of Stp1 was discovered by whole genome sequencing between the VISA isolate and its parental VSSA isolate from a single patient with endocarditis (Passalacqua et al., 2012). The two amino-acid insertion mutation may lead to the inactivation of Stp1 activity. The complementation of the VISA isolate with a wild copy of Stp1 reduced the vancomycin MIC (Passalacqua et al., 2012). Comparative genomics of a collection of VSSA/VISA isogenic pairs revealed the loss-of-function mutations in the *stp1* gene of VISA isolates. The construction of an *stp1* deletion mutant showed that Stp1 is involved in both vancomycin resistance and virulence (Cameron et al., 2012). A premature stop codon mutation and two other mutations were identified in a laboratory-derived teicoplanin-resistant *S. aureus* isolate. Complete genetic analysis revealed that the loss-of-function mutation in Stp1 contributed to teicoplanin and vancomycin resistance (Renzoni et al., 2011).

The transcriptome analysis of *stk1* deletion strains revealed that Stk1 can regulate many genes involved in nucleotide biosynthesis, cell wall metabolism, and autolysis (Donat et al., 2009). Several studies demonstrated that Stk1/Stp1 can cross talk with TCSs and other transcription factors (Table 3). VraR is a substrate of Stk1 (Ling et al., 2014); the phosphorylation of VraR at multiple sites (T106, T119, T175, and T178) by Stk1 negatively regulates its dimerization formation and DNA-binding activity, which can downregulate the VraSR system. Stk1/Stp1 can also regulate protein cysteine-phosphorylation of the SarA/MgrA family (SarA, MgrA, and SarZ) global regulators, which are involved in virulence and antibiotic resistance (Sun et al., 2012). In the *stp1* mutant, the phosphorylation state of SarA is increased. However, the binding to promoters is lost, thereby indicating that SarA phosphorylation attenuates its transcriptional activity. Another study revealed that CcpA is a substrate of the Stk1/Stp1 pair; the phosphorylation of CcpA (T18, T33) also decreased its DNA-binding activity (Leiba et al., 2012). By contrast, GraR can be phosphorylated by Stk1 at T128, T130, and T149 in the DNA-binding domain, but phosphorylation at these sites increased the DNA-binding activity of GraR (Fridman et al., 2013). Consequently, the expression of *dltABCD* operons is upregulated; these operons encode proteins to catalyze the d-alanyl esterification of the polyglycerol phosphate LTA and the polyribitol phosphate WTA (Fridman et al., 2013). The increased positive charge of LTA and WTA play important roles in exclusion of the cationic antibiotics such as vancomycin (Fridman et al., 2013). The stage V sporulation protein G (SpoVG) homolog of *S. aureus* modulates virulence factor synthesis and antibiotic resistance. The deletion of *spoVG* reduced teicoplanin and vancomycin resistance; although the level of resistance to teicoplanin was more pronounced than that to vancomycin (Schulthess et al., 2009). SpoVG can directly bind to the putative promoters of *lytN, femA*, and *lytSR*, and modulates oxacillin resistance in MRSA strain N315 (Liu et al., 2016). A recent study found that SpoVG is also subject to phosphorylation by Stk1; the Stk1-mediated phosphorylation markedly enhanced the DNA-binding activity of SpoVG (Bischoff et al., 2016). Therefore, the loss of *stp1* will cause the irreversible phosphorylation of several important transcription factors involved in vancomycin or teicoplanin resistance and virulence. Whether CcpE is regulated by Stk1/Stp1 awaits further investigation.

**Table 3:** **Summary of substrates of Stk1/Stp1 determined in *S. aureus***.

**Substrates**	**Function**	**Phosphorylation site(s)**	**Effects of phosphorylation**	**References**
VraR	Vancomycin-resistance-associated response regulator VraR	Thr106, Thr119, Thr175, Thr178	Decreased DNA-binding properties	Canova et al., 2014
SarA,	SarA/MgrA family transcriptional regulators	Cys9	Decrased DNA binding activity	Sun et al., 2012
MgrA,	SarA/MgrA family transcriptional regulators	Cys12	Decrased DNA binding activity	Sun et al., 2012
SarZ	SarA/MgrA family transcriptional regulators	Cys13	Decrased DNA binding activity	Sun et al., 2012
CcpA	Catabolite control protein A	Thr-18, Thr-33	Electrophoretic mobility shift assays demonstrated that the CcpA DNA binding activity was completely abrogated for the phosphorylated CcpA	Leiba et al., 2012
GraR	a two-component system involved in resistance to cationic antimicrobial peptides	Thr128, Thr130, Thr149	Increased DNA binding activity	Fridman et al., 2013
SpoVG	Modulator of virulence factor synthesis and antibiotic resistance	Thr4, Thr13, Thr24, Ser41	Enhanced the DNA binding activity	Bischoff et al., 2016
LuxS	Autoinducer-2 synthase	Thr14	The enzymatic activity of the phosphorylated isoform of LuxS was abrogated compared to that of non-phosphorylated LuxS	Cluzel et al., 2010
PurA	Adenylosuccinate synthase involved in synthesis of AMP	Not determined	Decreased enzymatic activity of PurA	Donat et al., 2009

Metabolomic studies found that the levels of cell wall synthesis precursors were significantly changed in the *S. aureus stk1* or *stp1* mutant (Liebeke et al., 2010). For instance, the levels of fructose-1,6-bisP, glucose-6-P, and fructose-6-P are significantly decreased in the *stp1* deletion mutant as compared with the wild type (Liebeke et al., 2010). This study indicated that metabolic enzymes might also be subjects for phosphorylation by Stk1. Stk1 can directly phosphorylate adenylosuccinate synthase (PurA) and downregulate its enzymatic activity (Donat et al., 2009). Autoinducer-2 (AI-2) is an important signaling molecule in quorum-sensing system of *S. aureus*, its synthase (LuxS) can be inactivated by Stk1 phosphorylation at T14 (Cluzel et al., 2010). The substrates of Stk1/Stp1 in *S. aureus* are summarized in Table 3.

#### Mutations in genes encoding proteolytic proteins

ClpP is a proteolytic subunit of the ATP-dependent Clp protease. A core proteolytic chamber is formed by ClpP and flanked by several ATPases, including ClpC, ClpX, ClpB, and ClpL. The ATPases determine substrate specificity and perform chaperon activities. The ClpP and Clp ATPases are involved in stress response, virulence, metabolism, and antibiotic resistance (Frees et al., 2014).

A 144 bp deletion of *clpP* caused the loss-of-function of ClpP with the WalK Q371 single amino acid deletion, which was found to mediate vancomycin resistance in a laboratory-derived VISA isolate (Shoji et al., 2011). The deletion of ClpP affects the expression of several important regulatory genes, including *agr, sigB, sarT*, and *walKR* (Shoji et al., 2011). Proteomics has revealed that numerous proteins are substrates of ClpC or ClpP (Feng et al., 2013; Graham et al., 2013), including proteins involved in cell wall synthesis, hydrolysis, and transcriptional regulation (Table 4). The cell wall synthesis proteins, such as PBP2, FemA, and FemB, and the autolysins, including Sle1 and Atl, can be hydrolyzed by ClpP; however, accumulated autolysins are catalytically inactive in the ClpP mutant, and the underlying mechanisms remain elusive (Feng et al., 2013). Transcription factors, such as CcpA, HprK (co-repressor of CcpA kinase), and CodY, are also substrates of ClpP or ClpC in *S. aureus* (Frees et al., 2014). The effects of proteolytic proteins, such as ClpP, on vancomycin resistance in VISA may occur via their regulational roles on the transcription factors. By contrast, ClpC, ClpP, and ClpB can also be repressed by the GraSR system (Falord et al., 2012b). However, ClpB is activated by the WalKR system, whereas ClpC is repressed (Delauné et al., 2012).

**Table 4:** **Summary of ClpP or ClpC substrates related to vancomycin resistance in VISA**.

**Protein name**	**Function**
Sle1	Autolysin precursor
Atl	Bifunctional autolysin precursor
Glck	Glucokinase
GlmS	Glucosamine–fructose-6-phosphate aminotransferase, isomerizing
GlmM	Phosphoglucosamine mutase
MurC	UDP-N-acetylmuramate–alanine ligase
MurG	UDP-glucose diacylglycerol glucosyltransferase
MurI	Glutamate racemase
MurE	UDP-N-acetylmuramoylalanyl-d-glutamate–2,6-diaminopimelate ligase
FemA	Formation of the pentaglycine cross bridge
FemB	Formation of the pentaglycine cross bridge
Pbp2	Penicillin-binding protein 2, peptidoglycan cross linking
SigA	RNA polymerase sigma factor
RpoA	DNA-directed RNA polymerase, alpha subunit
RpoB	DNA-directed RNA polymerase, beta subunit
CodY	GTP-sensing transcriptional pleiotropic repressor
SaeR	Response regulator SaeR
CcpA	Catabolite control protein A
AgrA	Staphylococcal accessory gene regulator A
RsbW	Serine-protein kinase RsbW

### Other genes involved in vancomycin resistance

The *airSR* genes encode an important TCS involved in cell wall biosynthesis and vancomycin resistance. The deletion of *airSR* showed reduced viability of *S. aureus* in the presence of vancomycin (Sun et al., 2013). The teicoplanin resistance factor A (TrfA) also affects vancomycin and oxacillin resistance (Renzoni et al., 2009). TrfA is a homolog of the MecA adaptor protein of the ClpC ATPase and is involved in the proteolysis. The transcription control of *trfA* is mediated by the thiol/oxidative stress global regulator Spx (Jousselin et al., 2013). The proteolytic degradation of Spx is governed by the YjbH protein, which is an adaptor protein of the ClpXP protease that was mutated in a laboratory-derived VISA isolate (Renzoni et al., 2011). Thus, the YbjH–Spx–TrfA cascade is important for vancomycin resistance in VISA isolates. The *tcaA* gene encodes a transmembrane protein, which is in the same operon as *tcaB* and *tcaR*. TcaB is a multidrug efflux pump, whereas TcaR is a transcriptional regulator of virulent determinants. The inducible expression of *tcaA* is dependent on VraSR (Chen et al., 2016). The inactivation of *tcaA* can increase the vancomycin resistance of *S. aureus* isolates (Maki et al., 2004).

## Conclusions and a proposed model

**Figure 3:**
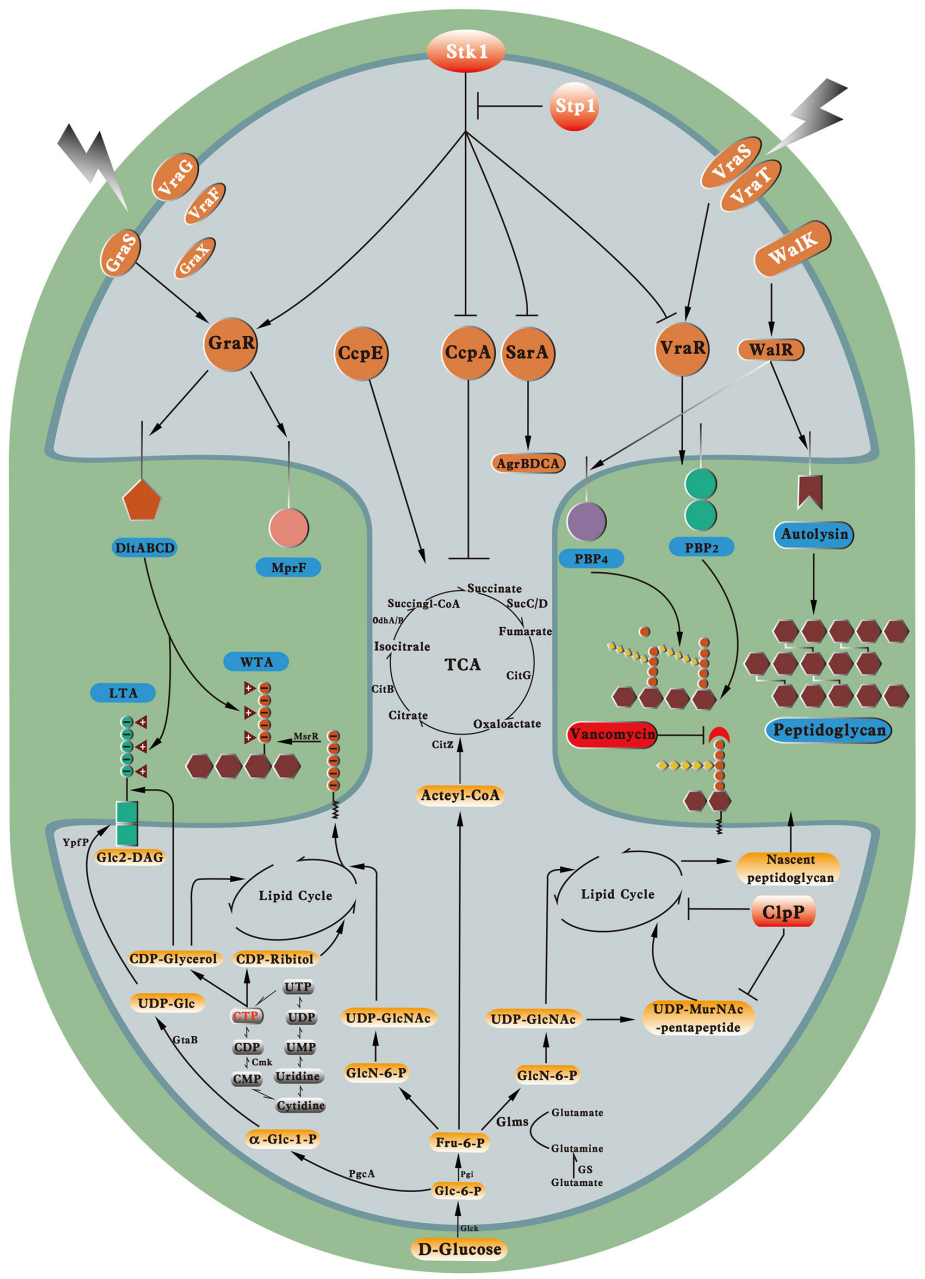
**Molecular events in VISA**. Key regulatory proteins and cell wall synthesis processes and their enzymes related to vancomycin resistance in VISA. WTA, wall teichoic acid; LTA, lipoteichoic acid; Glc, glucose; GlcN-6-P, glucosamine-6-phosphate; GlcNAc, N-acetylglucosamine; MurNAc, N-Acetylmuramic acid; DAG, diacylglycerol.

Given the increasing use of vancomycin, VISA emerged in the mid or late 1980s and has become a global health threat. Although the mechanisms for the promotion of vancomycin resistance in VISA are diverse, VISA exhibit several common characteristics, such as thickened cell walls, reduced autolysis, and attenuated virulence. The diverse mutations in certain genes identified in VISA imply the multiple complicated evolutionary pathways for VISA. We proposed a multiple-hit model for vancomycin resistance in VISA (Figure 3). On one hand, the mutated and constitutively activated VraTSR and GraSR systems increased the transcription of genes for cell wall synthesis and modification, including *pbpB, dltABCD*, and *mprF*, thereby increasing the cell wall biosynthesis and the d-alanyl esterification of LTA and WTA. The mutated and actively downregulated WalKR system decreased the expression of autolysins and PBP4, thereby decreasing autolysis and the muropeptide cross-linking in VISA. The mutation and loss of function in Stp1 led to irreversible phosphorylation of several transcriptional factors by Stk1, such as GraR, VraR, CcpA, and SarA, which will directly or indirectly influence vancomycin resistance in VISA. The mutation and loss of function in ClpP upregulated the transcription factors and cell wall synthesis proteins, such as PBP2, FemA, and FemB. Mutations occurred in the cell wall synthesis or hydrolysis genes, such as *msrR* and *sle1*, which could directly influence cell wall synthesis; mutations in the metabolic genes, such as *fdh2*, could adjust the cell metabolism to support cell wall synthesis precursors. These mutated genes collectively influenced the balance between cell wall synthesis and hydrolysis, which would lead to cell wall thickening. The thickening of the cell wall and its decreased cross-linking undoubtedly provide more free d-Ala–d-Ala residues. Thus, to arrive at the cell wall synthesis site, namely, the division septum, most of the vancomycin molecule might be trapped, thereby elevating the vancomycin resistance in VISA. On the other hand, the alteration of central metabolism (TCA) might enhance the lipid cycle in VISA by regulating the CcpE protein, which promotes the nascent peptidoglycan biosynthesis, as well as the synthesis of LTA and WTA. Mutations in metabolic genes, such as *cmk*, caused the downregulation of LTA and WTA synthesis as well as increased the vancomycin resistance in VISA.

## Author contributions

HP arranged the tables, QH wrote the manuscript, XR revised the manuscript.

## Funding

This work was supported by Natural Science grants 81471993 and 81672071 from National Natural Science Foundation of China.

### Conflict of interest statement

The authors declare that the research was conducted in the absence of any commercial or financial relationships that could be construed as a potential conflict of interest.
